# Host-pathogen interaction in arthropod vectors: Lessons from viral infections

**DOI:** 10.3389/fimmu.2023.1061899

**Published:** 2023-01-31

**Authors:** Nighat Perveen, Khalid Muhammad, Sabir Bin Muzaffar, Tean Zaheer, Nayla Munawar, Bojan Gajic, Olivier Andre Sparagano, Uday Kishore, Arve Lee Willingham

**Affiliations:** ^1^ Department of Biology, College of Science, United Arab Emirates University, Al-Ain, United Arab Emirates; ^2^ Department of Veterinary Medicine, College of Agriculture and Veterinary Medicine, United Arab Emirates University, Al-Ain, United Arab Emirates; ^3^ Department of Parasitology, University of Agriculture, Faisalabad, Pakistan; ^4^ Department of Chemistry, College of Science, United Arab Emirates University, Al-Ain, United Arab Emirates; ^5^ Department of Infectious Diseases and Public Health, Jockey Club College of Veterinary Medicine and Life Sciences, City University of Hong Kong, Kowloon, Hong Kong SAR, China

**Keywords:** immune system, innate immunity, virus circulation, haemocoel, haemocytes, antiviral defense

## Abstract

Haematophagous arthropods can harbor various pathogens including viruses, bacteria, protozoa, and nematodes. Insects possess an innate immune system comprising of both cellular and humoral components to fight against various infections. Haemocytes, the cellular components of haemolymph, are central to the insect immune system as their primary functions include phagocytosis, encapsulation, coagulation, detoxification, and storage and distribution of nutritive materials. Plasmatocytes and granulocytes are also involved in cellular defense responses. Blood-feeding arthropods, such as mosquitoes and ticks, can harbour a variety of viral pathogens that can cause infectious diseases in both human and animal hosts. Therefore, it is imperative to study the virus-vector-host relationships since arthropod vectors are important constituents of the ecosystem. Regardless of the complex immune response of these arthropod vectors, the viruses usually manage to survive and are transmitted to the eventual host. A multidisciplinary approach utilizing novel and strategic interventions is required to control ectoparasite infestations and block vector-borne transmission of viral pathogens to humans and animals. In this review, we discuss the arthropod immune response to viral infections with a primary focus on the innate immune responses of ticks and mosquitoes. We aim to summarize critically the vector immune system and their infection transmission strategies to mammalian hosts to foster debate that could help in developing new therapeutic strategies to protect human and animal hosts against arthropod-borne viral infections.

## Introduction

Insecta is the most abundant group of terrestrial animals, both in terms of numbers as well as species (approx. 5.5 million) (1). However, most insect species are not described (1, 2); about 80% of species yet to be identified (2). Insects provide many essential services in the natural ecosystem as pollinators and biological control agents, and help in nutrient recycling and food resources (3). However, many insect species are serious pests of cash crops or staple food crops. In addition, some insects (including mosquitoes, lice, fleas, and bed bugs), and ticks (Acari) serve as vectors transmitting a variety of pathogens (bacteria, viruses, nematodes and protozoa) to humans and animals (4). Among these, mosquito-borne and tick-borne viruses cause some of the most severe diseases with high fatality rates in humans and animals (5–7). In particular vector-borne viruses, including Zika, West Nile fever, Rift Valley fever, dengue, yellow fever, chikungunya, Japanese encephalitis, Crimean-Congo hemorrhagic fever, tick-borne encephalitis and Alkhurma hemorrhagic fever virus, are a continued threat to human and livestock health globally. Due to globalization and climate change, ticks and mosquitoes are occupying new geographic areas expanding the remit of vector-borne diseases.

Insects have an open circulatory system, the blood (haemolymph) mixes with the interstitial fluid and circulates in a body cavity called the haemocoel (8). Haemocytes or blood cells, the cellular components of haemolymph (9), are classified into various cell types such as prohemocytes, plasmatocytes, granulocytes, coagulocytes, oenocytoids, spherulocytes, thrombocytoids, and crystal cells (not all haemocyte types are present in most insects) (10, 11), with their main functions being immune response to pathogens including detoxification, coagulation, encapsulation and phagocytosis; phagocytosis; other cells are involved in carrying and transferring nutritive materials to various organs (12, 13). Viral transmission in insects is dependent on innate immune response (8). In this review, we discuss the innate immunity of arthropod vectors with specific reference to anti-viral immune responses in ticks and mosquitoes.

## Insect innate immune system and pathogen clearance mechanisms

**Figure 1:**
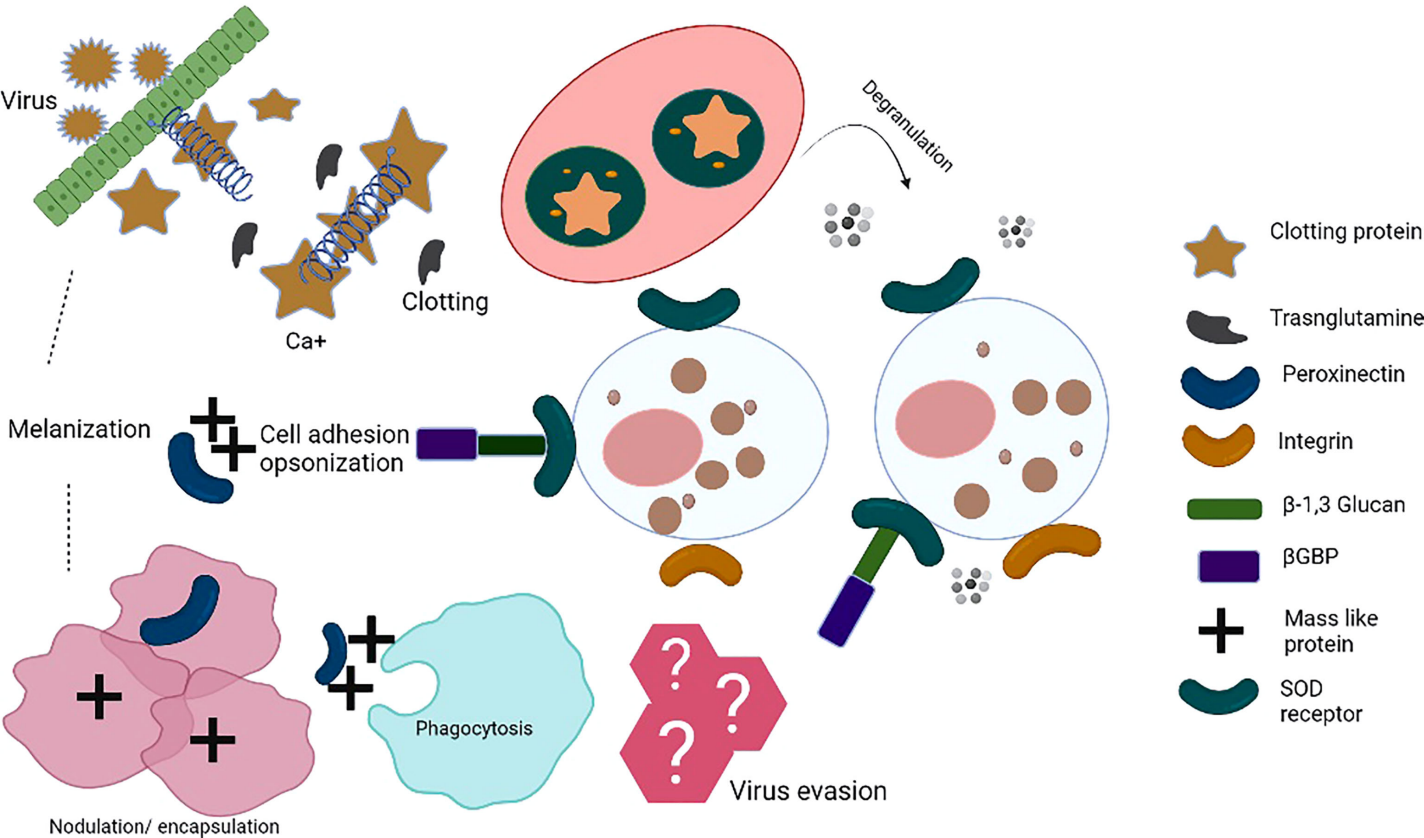
Insect innate immune mechanisms: When the insect’s immune system recognizes pathogen-associated molecular patterns (PAMPs) by pattern-recognition receptors (PRRs), pathogens encounter a complex system of humoral and cellular responses. Humoral responses include the production of antimicrobial peptides (AMPs), reactive nitrogen intermediates (RNI) or reactive oxygen intermediates (ROI), and a complex enzymatic cascade to regulate clotting or melanization. Cellular immune response involves various haemocytes that participate in phagocytosis/nodulation/encapsulation for pathogen clearance from haemolymph. In addition, different immune cells release toxic factors to kill pathogens, i.e. cell-mediated killing (complement components, etc.). These events take place in the haemocoel of the ticks. The series of immune reactions depict innate immunity. Clotting protein is a unique lipoprotein. Hemolymph clotting is induced upon transglutaminase (TGase) release from hemocytes/tissues. Calcium ions play an important role in the cascade leading to coagulation. TGase is involved in the coagulation, disturbing the chemical nature of the target pathogens. Peroxinectin is an opsonin that does attachment, spreads, and induces degranulation. SOD produces H_2_O_2_ from which hypohalic acid is released, which is a toxic substance, Prophenoloxidase activating system (proPO system) is an efficient part of the innate immune response. bGBP is a pattern recognition protein known to bind β-1,3, glucan. Masquerade (Mas-) like protein is also a multifunctional innate immune protein, an opsonin-like peroxinectin, capable of doing pathogen binding, inducing degranulation, and pathogen clearance. TGase, transglutaminase; Ca^2+^, Calcium, SOD, superoxide dismutase; βGBP, glucan with β-1,3 glucan binding protein; Mas-like protein,-- Masquerade- like protein.

Insect antiviral innate immunity has mostly been studied in *Drosophila melanogaster* (14). The innate immune system of insects acts through cellular and humoral components (15, 16), which together coordinate against bacterial and viral infections. Cellular immune responses include phagocytosis, nodulation, and encapsulation of pathogens by haemocytes (11, 17). However, during humoral response, pattern-recognition receptors (PRRs), which are germline-encoded, recognize pathogen-associated molecular patterns (PAMPs) and damage-associated molecules patterns (DAMPs). PAMPs include bacterial lipopolysaccharide (LPS), peptidoglycan, and fungal β-1,3-glucans (15). PRRs bind to the PAMPs and initiate opsonization of pathogens. The activation of downstream signaling induced by PRRs leads to the synthesis and secretion of effector molecules, for example reactive oxygen species (ROS), antimicrobial peptides (AMPs), and components of the phenoloxidase cascade; these effector molecules restrict infections and clear the intruding pathogens (8, 18–20). Blood sucking insects such as mosquitoes acquire various pathogens during blood feeding and their midgut epithelial cells act as the first line of defense and produce ROS and several AMPs. The expression of genes coding for AMPs depends on various signaling pathways such as Toll, Immune Deficiency (IMD), and Janus Kinase and Signal Transducer and Activator of Transcription (JAK/STAT) pathways; activation of these pathways inhibits viral replication (8, 21, 22). PRRs detect microbial invaders and initiate signaling cascades that hinder their proliferation in the host. This leads to engagement of adaptor molecules establishing multi-protein complexes comprised of kinases, transcription factors, and other regulatory molecules (23). **Figure 1** shows the complex interplay of insect innate immune responses.

Viral replication may be limited by activation of above-mentioned pathways. However, RNA interference (RNAi) is considered the most potent antiviral defense mechanism; it hinders viral replication yielding small RNAs (using viral double-stranded RNA as a template) targeting viral RNA degradation (24). Thus, innate immune system works through various effector mechanisms including phagocytosis, encapsulation, melanization, nodulation, lysis, RNAi, autophagy, and apoptosis.

### Phagocytosis of pathogens by haemocytes

Phagocytosis by haemocytes starts when PRRs such as thioester-containing proteins, Nimrod proteins, β-integrins, and peptidoglycan recognition proteins (PGRPs) bind PAMPs (25, 26). For instance, PGRP-LC initiates the phagocytosis of *E. coli* in *D. melanogaster* (26). The pathogen is then taken into a membrane-delimited phagosome. The phagosome fuses with a lysosome (phagolysosome), where the pathogen is digested by hydrolytic enzymes such as lysozyme, proteases, lipases, nucleases, and glycosylases (25). There are haemocytes that circulate in the haemolymph (circulating haemocytes) and tissue-resident haemocytes (sessile haemocytes) (13, 27). Insects have morphologically and functionally distinct haemocyte subpopulations. The classification of haemocytes has not been standardized across Insecta (25); however, the majority of haemocytes are phagocytic (25). In Lepidopterans and Hemipterans, the phagocytic haemocytes are granulocytes, whereas in fruit flies, plasmatocytes show phagocytic activities (13, 27). In mosquitoes, circulating and sessile haemocytes initiate phagocytosis within seconds of making contact with the pathogens (25, 27) and can ingest hundreds of bacteria in a short time.

### Encapsulation

Encapsulation is the most common type of defense reaction against parasites. Haemocyte-mediated encapsulation occurs in insects when large pathogens enter the haemolymph, for example, the eggs or larvae of parasites, protozoa and nematodes (25). Encapsulation in insects is of two types: cellular and humoral encapsulation. Haemocytes may or may not be involved in the humoral encapsulation that is associated with phenoloxidase; however, the cellular process may occur without melanization (11). In *Galleria mellonella*, during encapsulation, granulocytes release the adhesion protein, peroxinectin. It is a multifunctional molecule which is not only used for attachment and spreading but it also enhances encapsulation, degranulation, opsonization and peroxidase activity (28). Thus, this adhesion helps plasmatocytes attach to the layer of granulocytes which is then enclosed by several layers of plasmatocytes, followed by additional granulocytes (11, 25). Multiple layers of plasmatocytes surrounding the pathogen produce a capsule of multi-layered overlapping cells to sequester pathogen. In *Drosophila*, lamellocytes may commonly be observed in capsules; however, in Lepidoptera, both granulocytes and plasmatocytes are commonly observed in capsules (11, 16). In some cases, accompanied by encapsulation, plasmatocytes deposit pigment around the parasite (melanization). These defense responses, either alone or together, successfully kill parasites (29). In mosquitoes, the complement C3-like protein (AgTEP1) induces an immune response against *Plasmodium berghei*. Parasite death occurs when protein binds to the surface of the parasite and initiatesencapsulation by haemocytes (30).

### Melanization

Melanization is an immune effector mechanism that kills pathogens including bacteria, protozoa, and nematodes; it is also involved in wound healing (25). In melanization reaction, tyrosine converts to melanin precursors and then proteins cross-link to form a melanin layer that impounds an attacking pathogen and establishes a dark proteinaceous capsule. This can cause starvation or oxidative damage resulting in the pathogen’s death (31). Furthermore, this process also facilitates clearing of dead microbes. Melanization occurs with the coordination of PRRs, serine proteases, serine protease inhibitors, and enzymes and starts when PRRs (for example β-1,3 glucan, C-type lectins and Gram-negative binding proteins) recognize PAMPs and initiate a serine protease cascade. Phenoloxidase begins the production of melanin by hydroxylation of tyrosine. Many enzymes and PRRs involved in the melanization process are produced by haemocytes, for instance, oenocytoids (which are large and oval cells, their cytoplasm contains agglomerates of microtubules) (32) are the main producers of prophenoloxidase. Prophenoloxidase (ProPO) participates in melanization (25, 33). Therefore, haemocytes play an important role in clearing microbes from the insects’ haemolymph by forming melanotic capsules (29). Recently, the activity of the phenoloxidase/melanization responses was investigated in *Drosophila;* it was found that injection of Zika virus into wild-type adult flies caused melanin formation at the injection site by increasing phenoloxidase activity in the hemolymph (34).

### Nodulation

The nodulation process involves coordinated adherence of haemocytes in order to enclose large clusters of pathogens followed by melanization (25). In the case of bacterial infections, several haemocytes attach to the aggregates of microbes. This helps in the removal of a considerable number of bacteria from insects’ haemolymph through phagocytosis (11). Thus, haemocytes bind together to form a capsule around the pathogen (25) and nodulation is accomplished by the stimulation of prophenoloxidase and mature nodules’ melanization (11).

### Lysis

Lysis is immune-based disruption of the cellular membrane to kill the pathogens. This mechanism is difficult to observe because an immune-based reduction in infection intensity leads to pathogen death that is not an easily visible immune phenotype as compared to phagocytosis, encapsulation, nodulation, and melanization where pathogens can be seen inside haemocytes. Factors such as AMPs induce pathogen death through lysis (25). The AMPs include defensin and defensin-like peptides, cecropin and cecropin-like peptides, attacins and gloverins, and lebocins mostly detected in Diptera, Coleoptera and Lepidoptera and act against bacteria and fungi. Immune signaling pathways govern the production of AMPs. For example, in *D. melanogaster*, activation of the Toll pathway induces the transcription of drosomycin while activation of the Imd pathway induces the transcription of diptericin (35). Lysozymes are also involved in lytic activity. ROS and RNS (reactive nitrogen intermediates) affect lytic activity in the extracellular environment. Furthermore, reactive species are also involved in the antimicrobial response in the haemocoel (35, 36).

### RNA interference

RNAi is a process in which RNA molecules inhibit gene expression or translation by neutralizing targeted mRNA molecules; it is considered an ancient gene silencing pathway connected to antiviral defense (37). Small RNA-guided antiviral immunity was first revealed in plants, and subsequently, in fruit flies (*D. melanogaster*) and round worms (*Caenorhabditis elegans*) (37). By studying the function of insect genes, RNAi could be used for insect pest management (38). Among various defenses, activation of the RNAi pathway is the key antiviral mechanism in mosquitoes that leads to viral RNA degradation and replication inhibition (8, 39). The production of small RNAs from long viral double-stranded RNA (dsRNA) has a major role in the RNAi pathway and small RNAs include small interfering RNAs (siRNAs), microRNAs (miRNAs), and PIWI-interacting RNAs (piRNAs) (40). In the small interfering RNA (siRNA) pathway, a ribonuclease, Dicer-2 (Dcr2) cleaves the viral double-stranded RNA (dsRNA) to generate viral siRNAs (41). siRNA leads the Argonaute-2 (Ago-2) protein to target viral RNAs to initiate degradation (41). Ago-2 is a slicer protein and slicer activity is crucial for an effective RNAi response. Furthermore, virus replication is negatively controlled by the transcription of Vago, a cysteine-rich polypeptide that is activated by the binding of viral RNA to Dcr2 (42). In mosquitoes, the PIWI-interacting RNA pathway (piRNA) is also used in antiviral response in addition to the siRNA pathway (39, 43). piRNAs are small non-coding RNAs that interact with proteins, for instance, PIWI, Ago-3, and Aubergine (Aub) to form the piRNA-induced silencing complex (piRISC) and regulate RNA silencing (44). However, piRNA biogenesis seems to vary considerably between germline and somatic cells. Virus-derived piRNAs have been shown to be produced in whole *Aedes* mosquitoes upon infection with chikungunya and dengue virus (43).

### Apoptosis

Apoptosis, the programmed cell death, is characterized by distinct morphological features and energy-dependent biochemical mechanisms which is essential for proper development and functioning of the immune system and normal cell turnover (45). A range of cellular anti-viral mechanisms exist in insects, including incompatibility of viruses with hosts, apoptosis, and shutdown of protein synthesis (1). For example, certain species of Lepidoptera respond to viral infections by inducing apoptosis in infected cells (46). There are two major apoptotic pathways: the intrinsic pathway that is mediated by mitochondria, and the extrinsic pathway mediated by death receptors [CD95, TRAIL-R1 (TNF-related apoptosis-inducing ligand-R1) or TRAIL-R2] activated by their natural ligands, the TNF family. The caspases (cysteine aspartyl-specific proteases) activate in both pathways which cleave cellular substrates leading to the biochemical/morphological changes that cause cell death and inflammation. These two pathways may be linked and influenced by the molecules of one another (47). In *Drosophila*, apoptosis occurs following intrinsic pathway activated by intracellular signals such as shutdown of protein or mRNA production, or DNA damage, and involves the formation of large complexes at the mitochondrial membrane in the case of baculovirus (46).

### Autophagy

Autophagy is the process employed for degradation of intracellular materials and elimination of intracellular pathogens such as bacteria and viruses (48–50). Bacteria can be eliminated by autophagy in *Drosophila*. During this process, peptidoglycan recognition protein LE (PGRP-LE) on haemocytes recognizes the bacterial peptidoglycan and induces LC3/Atg8 proteins targeting autophagy to clear the infection of *Listeria monocytogenes* (51). Furthermore, autophagy is employed in the immune reaction against Rift Valley fever virus (52).

### Coagulation

In case of injury, an insect’s cuticle serves as the first line of defense, providing a physical and chemical barrier. The open circulatory system of arthropods has efficient mechanisms that can prevent haemolymph loss in case of injury and also trap microbes before their entry and spreading in the body cavity/hemocoel. Haemolymph clotting is of significance in the innate immune system, which has been studied in arthropods, crayfish and horseshoe crab. In the lepidopteran species, there are four steps in clotting system (28) First, haemocyte degranulation establishes extracellular aggregates that seal the wound and makes a soft clot. Then, initiation of the prophenoloxidase cascade/tranglutaminase (TGase) facilitates crosslinking to form the hard clot. Subsequently, plasmatocytes induce scab formation by spreading and sealing the clot from the haemocoel. Finally, epidermis regenerates, grows and replaces the scab (15).

## Arthropod vectors, viruses, and innate immune responses

### Mosquitoes

**Figure 2:**
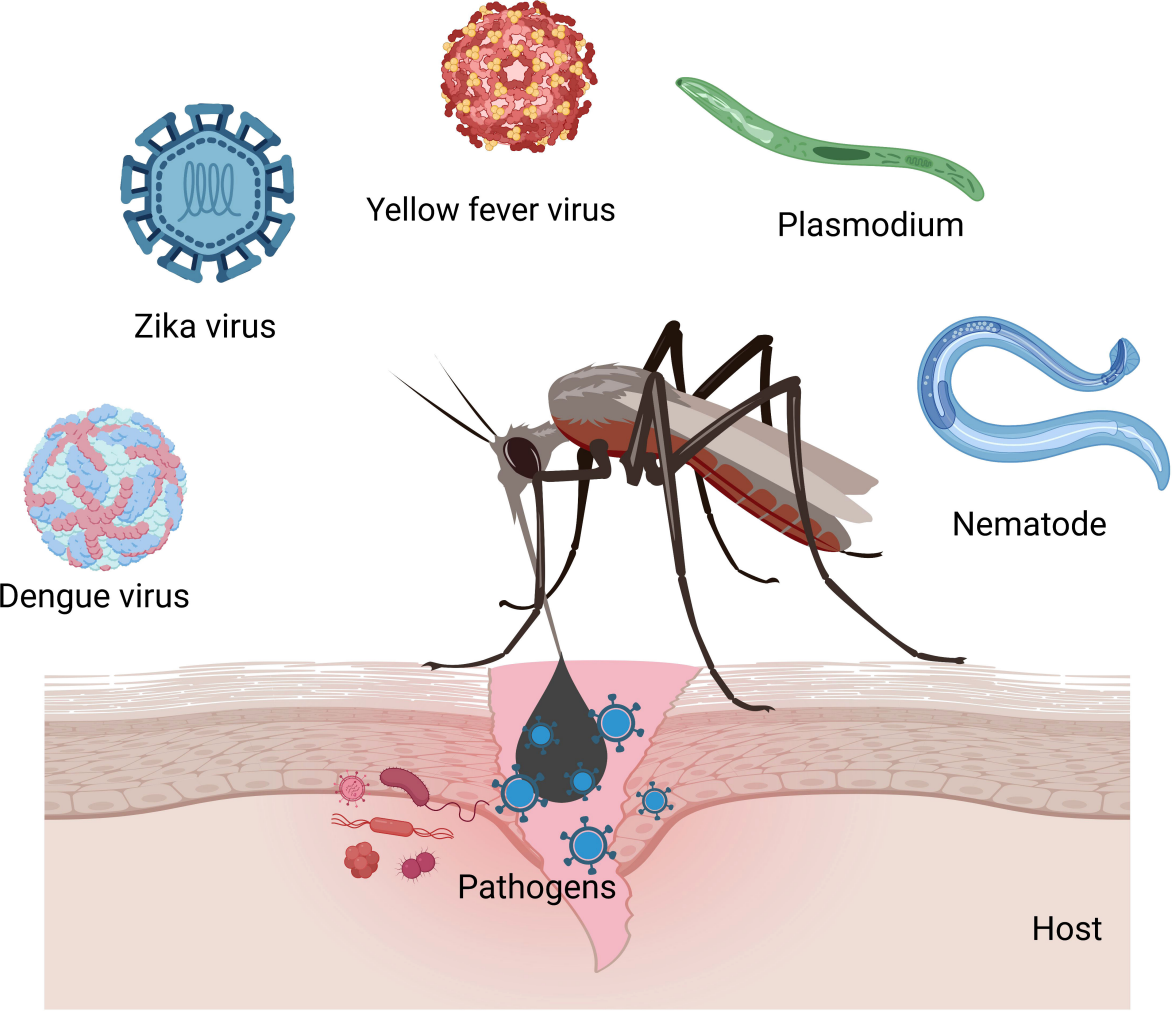
Mosquitoes transmit viruses (Zika, yellow fever, chikungunya, dengue, and West Nile), nematodes (filariasis), and *Plasmodium* parasites (malaria) in humans.

Mosquitoes (Diptera: Culicidae) are the vectors of pathogens that cause dengue, malaria, Japanese encephalitis, and filariasis (53). Out of 3000 documented mosquito species worldwide, more than 100 species are known to transmit infections to humans (54) (**Figure 2**). Mosquito-borne diseases are prevalent worldwide and infect over 0.7 billion population annually (54). Immune responses in vectors and vaccine strategies have been studied to circumvent the disease outbreaks (55, 56).

Most mosquito-borne viruses are RNA viruses, which belong to families Flaviviridae, Togaviridae, and Bunyaviridae (**Table 1**). Dengue virus is a member of the family Flaviviridae that causes 390 million infections every year (8). Furthermore, other members of the Flaviviridae such as Zika virus, West Nile virus, yellow fever virus, and Japanese encephalitis virus are also a cause of global concern due to increasing incidence and geographic expansion (64). In addition, their circulation poses serious health threats, for example, Zika virus has been linked to Guillain-Barré syndrome in adults and birth defects (microcephaly in prenatally infected infants) (65).

**Table 1:** Mosquito-borne viruses.

Family	Genus	Virus	Vector	Reference
Flaviridae	*Flavivirus*	Dengue virus	*Aedes aegypti*	(57)
	*Flavivirus*	Zika virus	*Aedes* spp.	(7)
	*Flavivirus*	West Nile virus	*Culex* spp.	(58)
	*Flavivirus*	Yellow fever virus	*Aedes* spp.	(57)
	*Flavivirus*	Murray Valley encephalitis virus	*Culex annulirostris*	(59)
	*Flavivirus*	Japanese encephalitis virus	*Aedes* spp.	(57)
	*Flavivirus*	St. Louis encephalitis virus	*Culex* spp.	(60)
Togaviridae	*Alphavirus*	Chikungunya virus	*Aedes* spp.	(61)
	*Alphavirus*	Venezuelan equine encephalitis virus	*Aedes* spp.	(61)
	*Alphavirus*	Semliki Forest virus	*Aedes* spp.	(62)
	*Alphavirus*	O’nyong nyong virus	*Anopheles* spp.	(63)
Bunyaviridae	*Orthobunyavirus*	La Crosse virus	*Aedes triseriatus*	(61)
	*Phlebovirus*	Rift Valley fever virus	*Aedes* spp.	(61)

**Figure 3:**
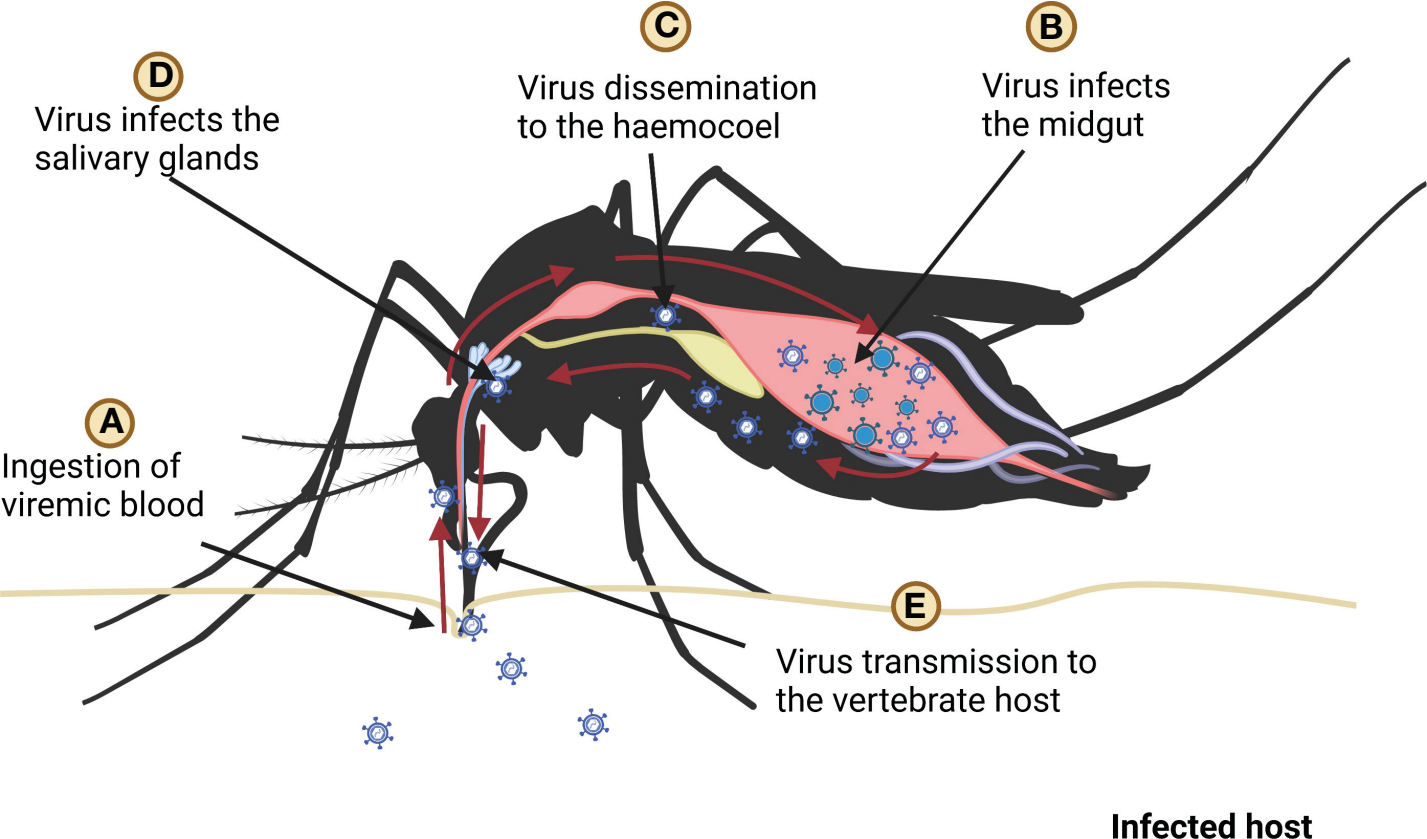
Ingestion, replication, and dissemination of virus in the mosquito, **(A)** mosquito ingests the blood of infected host, **(B)** ingested virus infects the midgut **(C)** after replication in the midgut virus spreads in the haemocoel, **(D)** after dissemination virus infects salivary glands **(E)** finally mosquito transmits virus to host by biting it.

In mosquitoes, virus enters the midgut when it ingests the infected blood meal. The antiviral defense mechanism of mosquitoes is triggered as the virus is recognized by PRRs; however, the dissemination of the virus to the salivary glands is poorly understood (8). It is assumed that when the virus enters midgut epithelium, it replicates in the tissue and subsequently it gets disseminated to the haemocoel (66). The virus may spread through the haemolymph circulation to other tissues/organs including salivary glands, trachea, and neural tissues (21, 67) (**Figure 3**). The translocation of the virus to the salivary glands is crucial for their transmission to vertebrate hosts (68, 69).

In mosquitoes, during viral infection, PRRs recognize virus-conserved PAMPs to instigate immune responses. Degradation and thereby inhibition of viral replication occurs through activation of the RNAi pathway which is the main antiviral mechanism (70). Despite antiviral defense mechanisms that hinder/limit viral replication, the immune system of the mosquito may not be able to effectively clear the virus (8, 71). Without significant lethal effects of viral infection to hosts (mosquitoes), mosquitoes tolerate/resist long-lasting infections that make them efficient vectors for the emergence or re-emergence of viral diseases. Therefore, to conceptualize anti-vectorial strategies, understanding the mechanisms of mosquito tolerance/resistance to viral infection is vital. Furthermore, we can develop novel anti-vector strategies such as use of genetically modified mosquitoes (72–76) and *Wolbachia*-infected mosquitoes (77–80) for resistance to viral pathogens to prevent human infections through understanding of the innate immune responses of mosquitoes (8). In 2021, in United States, first genetically engineered mosquitoes were released in a field trial after many years of fight for regulatory approval and its public acceptance for controlling populations of wild mosquitoes (*Aedes aegypti*), which carry viruses such as chikungunya, dengue, yellow fever, and Zika (81). *Aedes aegypti* was transinfected with the endosymbiont *Wolbachia pipientis* as a vector control strategy for dengue virus transmission. The *Wolbachia* infected mosquitoes showed a decreased biting success due to bendy proboscis (78), therefore limit virus transmission in populations. Furthermore, *Wolbachia*-harboring mosquitoes could reduce Zika virus transmission and can be effective vector control strategy (79).

### Ticks

Ticks are responsible for the transmission of a variety of pathogens that cause diseases in humans and animals (82–84), including various arboviruses (85–87). However, the majority of human viral diseases transmitted by tick vectors are caused by flaviviruses, while other tick-borne viruses belong to other families such as Asfarviridae, Bunyaviridae, Flaviridae, Orthomyxoviridae, and Reoviridae (88) (**Table 2**). Tick-borne viruses are mostly RNA viruses that replicate in ticks as well as in vertebrate cells (86); however, antiviral innate immune mechanism is yet to be explored.

**Table 2:** Tick-borne viruses.

Family	Genus	Virus	Vector	Reference
Flaviridae	*Flavivirus*	Alkhurma hemorrhagic fever virus	*Ornithodoros savignyi *	(89)
	*Flavivirus*	Kyasanur Forest disease virus	*Haemaphysalis* spp.	(57)
	*Flavivirus*	Tick-borne encephalitis virus	*Ixodes* spp.	(57)
	*Flavivirus*	Powassan virus	*Ixodes* spp.	(90)
	*Flavivirus*	Omsk haemorrhagic fever	*Dermacentor* spp.	(91)
	*Flavivirus*	Louping ill	*Ixodes* spp.	(92)
	*Flavivirus*	Kadam virus	*Hyalomma dromedarii*	(93)
	*Flavivirus*	Langat virus	*Ixodes* spp.	(94)
Bunyaviridae	*Phlebovirus*	Heartland virus	*Amblyomma* spp.	(95)
	*Phlebovirus*	Severe fever with thrombocytopenia syndrome virus	*Haemaphysalis* spp.	(96)
	*Phlebovirus*	Uukuniemi virus	*Ixodes* spp.	(97)
	*Nairovirus*	Crimean-Congo haemorrhagic fever virus	*Hyalomma* spp.	(5)
	*Nairovirus*	Nairobi sheep disease virus	*Rhipicephalus appendiculatus*	(5)
Orthomyxoviridae	*Thogotovirus*	Thogoto virus	*Rhipicephalus* spp.	(98)
Reoviridae	*Orbivirus*	Tribec virus	*Ixodes* spp.	(99)
	*Orbivirus*	Kemerovo virus	*Ixodes* spp.	(99)
	*Coltivirus*	Colorado tick fever virus	*Dermacentor andersoni*	(100)
Asfarviridae	*Asfivirus*	African swine fever virus	*Ornithodoros* spp.	(101)

After mosquitoes, ticks are the second most important arthropod vectors (102), feeding on the blood of various vertebrate hosts including birds, mammals, amphibians and reptiles (103). Tick feeding can cause anemia in vertebrate hosts as a single adult female hard tick is able to consume more than 1 mL of blood (104), thereby adversely impacting on livestock health and productivity.

**Figure 4:**
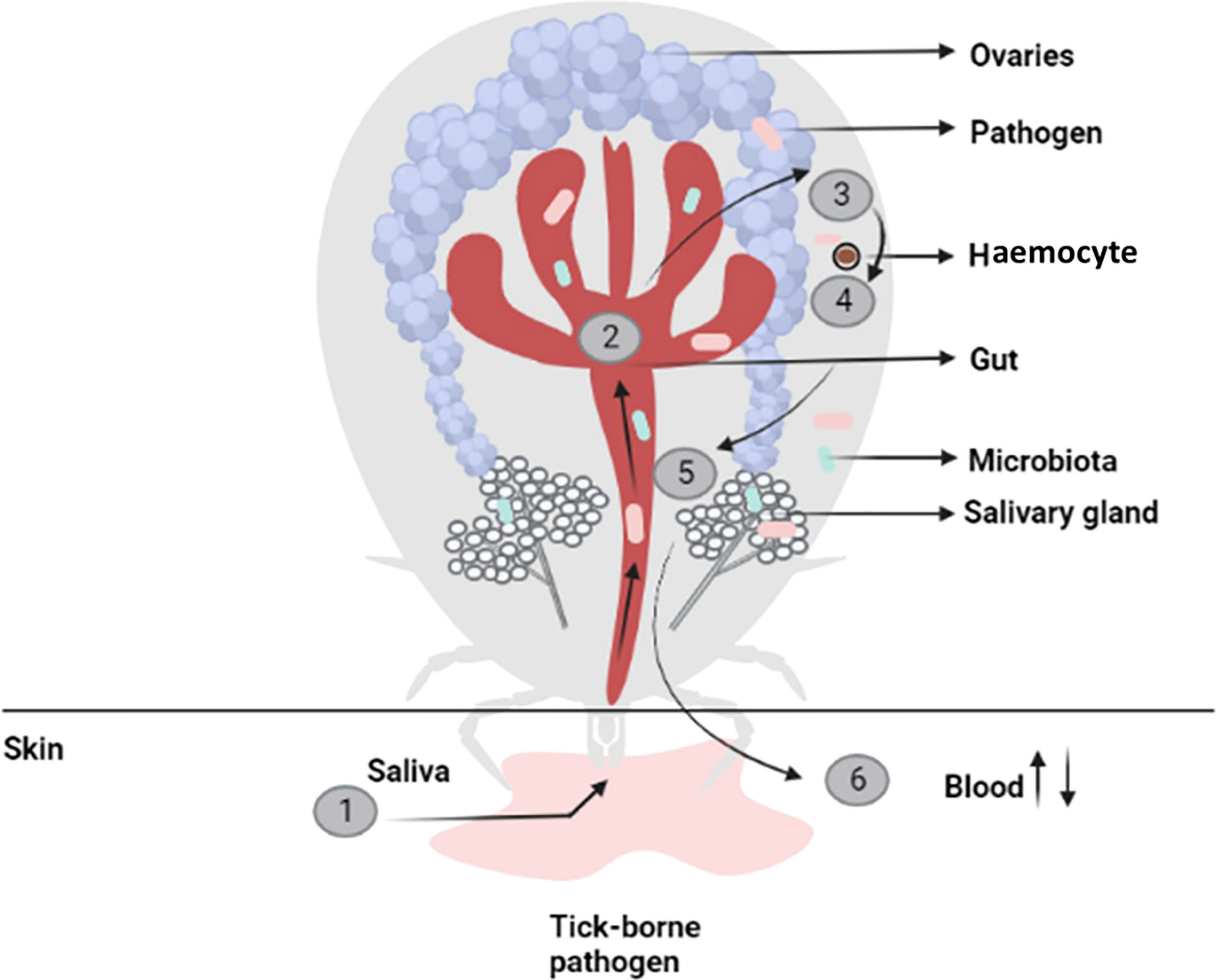
Acquisition, development, and transmission of pathogens by ticks. 1. When ticks bite, pathogens are ingested with the meal. 2. Pathogens either stay until the next meal or move through the gastrointestinal epithelium 3. The extraneous pathogen can potentially harm the tick’s body, depending on the type of pathogen. 4. Pathogens enter the salivary gland and attack the acini through the epithelium. 5,6. Pathogens, along with saliva, are introduced into a new host during feeding, disrupting host homeostasis and setting off inflammatory responses.

Tick feeding can also result in virus transmission. In the natural ecosystem, ticks become infected during viremic and non-viremic transmissions. In the case of viremic transmission, ticks become infected by feeding on the viremic vertebrate, whereas non-viremic transmission occurs when virus from an infected tick is transferred to an uninfected tick during co-feeding in a contained area on the skin of vertebrate host (88). During feeding, ticks secrete salivary molecules (105) that contain a mixture of proteins, peptides and non-peptide molecules that modulate host hemostasis and immune responses (106). Tick saliva can facilitate the transmission of viruses and other pathogens to the host during blood feeding by modulating host immune response (107). However, how pathogens evade different protective pathways and persist in the vector need to be explored. Tick-borne pathogens such as *Anaplasma*, *Borrelia burgdorferi sensu lato*, *Ehrlichia*, *Francisella*, and relapsing fever spirochetes have evolved various immune evasion strategies such as altering surface components, complement inhibition, antimicrobial molecule blocking, and inhibiting cytokines (108). Several tick-borne microbes modulate their outer-surface constituents via differential expression of various surface proteins through transcriptional regulation and intragenic recombination. Therefore, alteration of surface antigens allows microorganisms, for example, *B. burgdorferi* and relapsing fever spirochetes, to evade neutralizing host antibody response and encourage persistent infections in animals (109, 110). Surface components of bacteria may serve as PAMPs through recognition by TLRs (Toll-like receptors). Certain pathogens, for example *Francisella tularensis* mask their surfaces (to evade immune sensing) by synthesizing a carbohydrate-based capsule that inhibits the antibody and complement deposition on the cell wall, and provide protection against microbicidal host responses (for instance, opsonization) (111). Some bacteria, for example, *B. burgdorferi* and *Anaplasma* use host lipids (as a building block for the biogenesis of their membranes) that helps in bypassing host immune responses by avoiding the immune cells (108, 112). The cytokines play crucial roles in the integration of the innate and adaptive immune responses that are important for host defense against pathogens; various tick-borne microbes inhibit or enhance cytokine expression (108). For example, *Anaplasma phagocytophilum* infection leads to disruption of the IFN-γ (interferon-gamma) signaling pathways and downstream phagocytosis events by neutrophils (113). *Ehrlichia chaffeensis* may change early immune responses by inhibiting transcription of interleukin (IL)-12, IL-15, and IL-18 genes that allow *Ehrlichia* survival in macrophages (114). *Borrelia burgdorferi* or *A. phagocytophilum* infections trigger ticks (Ixodes) JAK/STAT or IMD pathways that stimulate a robust microbicidal response (115, 116). *Anaplasma phagocytophilum* activates the expression of tick antifreeze glycoprotein (IAFGP), which enhances the cold tolerance and survival of *Ixode scapularis* that eventually assist in persistence of pathogen in nature (117). Furthermore, *A. phagocytophilum* may modulate expression of salivary gland proteins (such as Salp16 and P11) from *I. scapularis* that aid its survival (118, 119). A pathogen ingested within the blood meal interacts with the tick gut (**Figure 4**) (104), colonizes the gut epithelial cells and/or crosses the gut epithelium to enter the haemocoel and may spread through haemolymph circulation to all tissues and organs (104). In haemolymph, complement-like molecules such as C3, C4, and C5 proteins opsonise pathogens that can be phagocytosed by haemocytes (30). Pathogens can also be destroyed by various types of effector molecules including AMPs, complement-like molecules, and factors of redox metabolism. Thereafter, the pathogen reaches the salivary glands to successfully transmit through saliva to the vertebrate host during the next blood feeding. Some pathogens, e.g. bacteria, also have the ability to invade tick ovaries and may transmit to progeny trans-ovarially (**Figure 4**). In the tick’s salivary glands, ovaries and midgut, pathogens have to deal with resident microorganisms as well as tick immune responses that impact the vector competence. Understanding the immune factors involved in interactions between ticks and tick-borne pathogens is essential to delineate the biology of tick-transmitted diseases and could help to detect targets for developing new strategies to block pathogen transmission (120). The acquisition, development, and transmission of diseases by ticks are explained in **Figure 4**.

To understand host-virus interaction, studies have been performed by injecting virus into *D. melanogaster* flies (121). This method has been shown to be relevant for identifying pathogen virulence factors and host defense mechanisms; however, injecting the virus amounts to bypassing the hosts’ natural protection barriers. Various studies have revealed that the transmission route used by pathogens has a substantial impact on the intensity of an infection and differential immune responses (122, 123). *Drosophila* and mosquitoes have a holometabolous life-cycle; they undergo metamorphosis between four life stages: egg, larva, pupa, and imago (adult), whereas ticks exhibit hemimetabolous development with life-cycles consisting of four stages: egg, larva, nymph and adult (103). The pathogen tropism and infection outcome may thus depend on the route of infection of the pathogen and the developmental stages of the arthropod (123, 124).

## Conclusion

Presently, treatments are available for many vector-borne viral diseases. However, vaccines are not available for the majority of mosquito- or tick-transmitted viral diseases. There is a focus on diagnosis, treatment and on vector control strategies to help prevent viral disease transmission and spread. In the case of mosquitoes, field tests are being conducted on the utility of genetically modified mosquitoes for control, however, there are concerns regarding the introduction of transgenic organisms into the wild. This approach is new and there is limited knowledge about how mosquito antiviral defense mechanisms may evolve with this strategy. The tick immune system and antiviral defense responses remain poorly understood. It is crucial to know how the mosquito and tick innate immune systems respond to viral infection and replication, and how the viruses are actually transmitted to a healthy mammalian host, which may help in the development of novel strategies to block or control vector-borne virus transmission. A continued integration of the expertise of ecologists, animal virologists, immunologists, entomologists, molecular biologists, geneticists, and public health personnel are required to achieve optimal health outcome for human, animals and the environment, and to eliminate the risk of these vector-borne diseases in the future.

## Author contributions

Conceptualization: NP, AW, and UK. Writing—original draft preparation: NP. Writing—review and editing: NP, UK, AW, KM, SM, OS, BG, and NM. Visualization: NP, TZ, UK, and KM. Supervision: AW. Project administration: AW. All authors contributed to the article, read and approved the final manuscript. All authors contributed to the article and approved the submitted version.
